# Intramuscular Administration of a Synthetic CpG-Oligodeoxynucleotide Modulates Functional Responses of Neutrophils of Neonatal Foals

**DOI:** 10.1371/journal.pone.0109865

**Published:** 2014-10-15

**Authors:** Noah D. Cohen, Jessica R. Bourquin, Angela I. Bordin, Kyle R. Kuskie, Courtney N. Brake, Kaytee B. Weaver, Mei Liu, M. Julia B. Felippe, Michael H. Kogut

**Affiliations:** 1 Equine Infectious Disease Laboratory, Department of Large Animal Clinical Sciences, College of Veterinary Medicine & Biomedical Sciences, Texas A&M University, College Station, Texas, United States of America; 2 Department of Clinical Sciences, College of Veterinary Medicine, Cornell University, Ithaca, New York, United States of America; 3 Food and Feed Safety Research, Agricultural Research Service, Southern Plains Agricultural Research Center, USDA, College Station, Texas, United States of America; Federal University of São Paulo, Brazil

## Abstract

Neutrophils play an important role in protecting against infection. Foals have age-dependent deficiencies in neutrophil function that may contribute to their predisposition to infection. Thus, we investigated the ability of a CpG-ODN formulated with Emulsigen to modulate functional responses of neutrophils in neonatal foals. Eighteen foals were randomly assigned to receive either a CpG-ODN with Emulsigen (N = 9) or saline intramuscularly at ages 1 and 7 days. At ages 1, 3, 9, 14, and 28, blood was collected and neutrophils were isolated from each foal. Neutrophils were assessed for basal and *Rhodococcus equi*-stimulated mRNA expression of the cytokines interferon-γ (IFN-γ), interleukin (IL)-4, IL-6, and IL-8 using real-time PCR, degranulation by quantifying the amount of β-D glucuronidase activity, and reactive oxygen species (ROS) generation using flow cytometry. *In vivo* administration of the CpG-ODN formulation on days 1 and 7 resulted in significantly (P<0.05) increased IFN-γ mRNA expression by foal neutrophils on days 3, 9, and 14. Degranulation was significantly (P<0.05) lower for foals in the CpG-ODN-treated group than the control group at days 3 and 14, but not at other days. No effect of treatment on ROS generation was detected. These results indicate that CpG-ODN administration to foals might improve innate and adaptive immune responses that could protect foals against infectious diseases and possibly improve responses to vaccination.

## Introduction

Foals are known to be especially susceptible to a variety of bacterial infections, including *Rhodococcus equi*, *Salmonella*, *Clostridium difficile* and *C. perfringens*, and a number of Gram-negative and Gram-positive organisms that are associated with sepsis [1]–[7]. Consequently, pneumonia, diarrhea, and sepsis are leading causes of death in young foals [8], [9]. Neonatal susceptibility to infectious diseases is attributable in part to their diminished immune responses. Because neonatal adaptive immune responses are relatively deficient and naïve, newborns rely largely on innate immune responses for protection against bacterial infections [10], [11].

Neutrophils are critical effector cells of innate immunity [11], [12]. Neutrophils are the most abundant innate immune cells in blood, and they play a role in linking innate and adaptive immunity by recruiting and activating cells of the innate immune response. Evidence exists that neutrophils play a protective role against pathogens affecting neonates [10], [11]. Our laboratory has demonstrated the importance of neutrophils in protecting mice against experimental infection with *R. equi* [13]. Moreover, foals with relatively lower circulating neutrophil concentrations at 2 and 4 weeks of age were significantly more likely to subsequently develop *R. equi* pneumonia than foals with higher concentrations [14].

As observed for adaptive immune responses, innate immune responses also may be diminished in neonates [10], [11]. Age-dependent impairment of neutrophil function contributes to neonatal susceptibility to infectious diseases [10], [11], [15]. Although several studies indicate that foal neutrophils function similarly to those of adults after accounting for the reduced opsonic capacity of young foals relative to adults [16]–[19], there is increasing evidence of deficiencies in functional responses of foals. Foal neutrophils have been demonstrated to have decreased bactericidal capacity concurrent with reduced serum opsonic capacity [20], [21]. Age-related changes in basal and *R. equi*-stimulated cytokine gene expression by foal neutrophils have been described [22]–[24]. If the ability of neutrophils to protect foals against infections is compromised during early life, enhancing the functional responses of neutrophils in newborn foals might be an effective preventive strategy because both innate and adaptive immune responses could be impacted by such an approach.

Recently, our laboratory has demonstrated that stimulation *in vitro* with a synthetic cytosine-phosphate guanine oligodeoxynucleotide (CpG-ODN) could significantly increase pro-inflammatory cytokine gene expression and degranulation of neutrophils collected from foals as young as 1 day of age [23], [24]. While exciting, these results do not indicate whether administration of CpG-ODNs *in vivo* can alter the functional responses of foal neutrophils. Thus, the purpose of the study reported here was to determine whether *in vivo* administration of CpG-ODNs intramuscularly (IM) in combination with an adjuvant to foals could stimulate functional responses of foal neutrophils evaluated *ex vivo*. The functional responses we investigated include production of reactive oxygen species (ROS), cytokine gene expression, and degranulation.

## Materials and Methods

### Ethics statement

All procedures for this study, including collection of blood samples and administration of treatments to foals, were reviewed and approved by the Texas A&M University Institutional Animal Care and Use Committee (protocol number AUP # 2008-167) and the Texas A&M University Institutional Biosafety Committee (permit number 2008-109-Cohen). The foals used in this study were owned by Texas A&M University, and permission for their use was provided in compliance with the Institutional Animal Care and Use Committee procedures.

### Foals

Twenty-two healthy Quarter Horse foals were used for this study. All foals were born healthy and had age-appropriate results of complete blood count (CBC) on day 2 of life, and adequate transfer of passive immunity as assessed by a commercially-available qualitative immunoassay for serum concentration of total IgG (SNAP Foal IgG test; IDEXX, Inc., Westbrook, ME, USA). All foals were monitored daily by Texas A&M University Horse Center staff for clinical signs of disease, and inspected at least twice weekly by a veterinarian for clinical signs of disease. All foals except 1 remained free of clinical signs of disease and in good health throughout the study. One foal sustained musculoskeletal trauma at approximately 2 weeks of age and was excluded from the study. The respective dams of foals were fed 6.4 kg per horse per day of a 13% horse pellet (crude protein: 13.5%; crude fat: 4.5%; crude fiber: 10%); the foals and their mares were allowed free access to coastal Bermuda grass hay, plus grazing of pastures at the Texas A&M University Horse Center where the mares were maintained.

The foals were assigned randomly to 1 of 2 experimental groups using a block design prior to birth. Foals in the control group (N = 8) received 2.5 ml of sterile 0.9% sodium chloride solution IM on days 1 and 7 after birth. Foals in the CpG group (N = 11; 1 foal excluded because of trauma that required extensive veterinary care) received 2.5 mg of CpG-ODN 2142 (generously provided by Merial, Ltd., Duluth, GA, USA) diluted in sterile saline in combination with 1 ml of a commercial emulsifier (Emulsigen, MVP Technologies, Omaha, NE, USA) for a total volume of 2.5 ml administered IM on days 1 and 7 after birth. The selection of CpG-ODN 2142 was based on prior *in vitro* evidence of the stimulatory effect of this CpG-ODN on foal leukocytes [24]. The CpG-ODN was combined with Emulsigen because there is extensive evidence that the combination of CpG-ODNs with Emulsigen improves the immunomodulatory effects of CpG-ODNs administered either when paired alone with Emulsigen or when combined with Emulsigen and specific antigens in vaccines [25]–[35]. On days 1, 3, 9, 14, and 28 after birth, approximately 60 ml of blood were collected into 8-ml tubes containing sodium heparin as an anticoagulant (N = 5 tubes), 8-ml tubes containing a clot activator (N = 2 tubes), and a 4-ml tube containing ethylenediaminetetraacetic acid (EDTA) as an anticoagulant; the latter tube was used for determining a complete blood count. For neutrophil isolation, whole blood was allowed to settle at room temperature for 30 to 45 min, and then the plasma and buffy coat layers were used for neutrophil purification as described previously [22]–[24]. Neutrophils were isolated by layering whole blood over a discontinuous Histopaque (Sigma-Aldrich, St. Louis, MO, USA) gradient (specific gravity 1.077 over specific gravity 1.119), and centrifuged (700×g, 30 min, room temperature). After centrifugation, the 1.119 band containing the neutrophils was removed and washed once with Hanks balanced salt solution (HBSS) and centrifuged (300×g, 10 min). The neutrophil concentration was determined using an automated cell counter (Cellometer Auto T4, Nexcelcom, Lawrence, MA, USA), and purified neutrophils were examined microscopically to determine purity (to confirm>98% of neutrophils in the cell preparations). Neutrophils were then resuspended in HBSS to a final concentration of 2×10^7^ cells/ml.

### Cytokine mRNA expression

Freshly isolated neutrophils (approximately 5×10^6^/well) were incubated in 1 ml RPMI medium, with or without stimulation using live, virulent *Rhodococcus equi* (ATCC strain 33701) at a modulus of infection of 10 bacteria to 1 neutrophil, in 12-well tissue culture plates at 37°C in 5% CO_2_ in triplicate. After 2 h of incubation, cells were harvested, and RNA extraction, DNase treatment, and cDNA synthesis were carried out as described previously [22]–[24]. Expression of mRNA of equine cytokines (IFN-γ, IL-4, IL-6, and IL-8) was measured using real-time quantitative RT-PCR. These cytokines were selected on the basis of results of *in vitro* effects of CpG-ODNs on foal neutrophils, ^23^ in particular that IL-6 and IL-8 are abundantly expressed by neutrophils; ^22-24^ IFN-γ was selected because it is the prototypic T helper 1-type cytokine and IL-4 is considered a prototypic T helper 2-type cytokine. Absolute quantification of gene copy number was conducted using the standard curve method. Standard curves were constructed for each gene, by cloning the corresponding equine cDNA gene sequence into a plasmid vector (IFN-γ, IL-4, IL-6, and IL-8) and transforming into chemically competent *E. coli*. Plasmid DNA was extracted, and the accuracy of the cloned sequence was confirmed through bi-directional sequencing. The copy number of the plasmid standard was calculated using the following formula: copies/µl = plasmid concentration (g/µl)×6.022×10^23^/plasmid length (bp)×660. A series of corresponding standards was prepared by performing 10-fold serial dilutions of plasmid DNA in the range of 100 million to 0.1 copies per PCR reaction. Gene copy numbers for each target gene were determined by comparison of the fluorescence generated by each sample with standard curves of know quantities. Primer and probe sequences for each gene tested were described in Table S1. Primers were designed to span 2 exons to avoid amplifying genomic DNA, and were further tested to document that they did not amplify genomic DNA by testing RNA preparations with and without residual genomic DNA prior to use. The PCR reactions were performed in optical 384-well plates using a 7900HT Fast Real-Time PCR System (Applied Biosystems, Foster City, CA, USA). Each 20-µl reaction mixture contained 10 µl of 2× TaqMan Universal Master Mix (Invitrogen, Life Technologies, Grand Island, NY, USA), 60 ng cDNA, 400 nM each of forward and reverse primers, and 200 nM of TaqMan probe (Applied Biosystems, Life Technologies, Grand Island, NY, USA) and molecular grade water. The thermal profile consisted of an initial hold at 50°C for 2 min, followed by a single denaturation step at 95°C for 10 min, and then 40 cycles of 95°C for 15 s, 60°C for 60 s. Samples were tested by PCR in duplicate for each reaction.

### Degranulation

Neutrophil degranulation was detected by quantifying the amount of β-D glucuronidase activity in the culture medium following stimulation of the neutrophils with opsonized *R. equi* (ATCC 33701); serum from a mare with a high optical density of anti-*R. equi* antibodies as determined by ELISA was used to opsonize the *R. equi*. *Rhodococcus equi* was selected as a stimulus on the basis of prior evidence of its effectiveness [22]–[24]. Neutrophils (1×10^7^ cells/ml) were incubated for 1 h on a rocker platform at 37°C with live, virulent *R. equi* at an MOI of 10 bacteria per neutrophil (as for cytokine expression). The cells were then centrifuged (250×g, 10 min, 4°C) and the resultant supernatants were used for the assay. A 25-µl aliquot of each supernatant was added to wells in a black flat-bottom ELISA plate and incubated with 50 µl of substrate (10 mM 4-methylumbelliferyl-β-D-glucuronide, 0.1% Triton X-100 in 0.1 M sodium acetate buffer) for 4 h at 37°C. The reaction was stopped by adding 200 µl of stop solution (0.05 M glycine and 5 mM EDTA; pH 10.4) to each well. Liberated 4-methylumbelliferone was measured fluorometrically (excitation wavelength of 355 nm and an emission wavelength of 460 nm) with a fluorometric plate reader (BioTek Synergy 2, Winooski, VT, USA). These values were converted to µmoles of 4-methylumbelliferone generated using a standard curve of known concentrations.

### ROS generation

To measure ROS production, whole blood leukocytes were isolated by layering whole blood onto Ficoll-Paque, using 2 parts blood and 1 part gradient. Erythrocytes were allowed to sediment at room temperature for 30 min. The upper layer was removed and washed once with 15 ml HBSS and centrifuged (300×g for 10 min, 4°C). Residual erythrocytes were lysed by the addition of NH_4_Cl (pre-warmed to 37°C) and incubated at room temperature for 3 min. Cells were centrifuged (300×g, 10 min, 4°C) and the resultant leukocyte pellet was washed once with HBSS and centrifuged (300×g, 10 min, 4°C). The leukocytes were re-suspended in 1 ml HBSS and kept on ice until aliquoted for assay.

A 10 mg/ml stock solution of dihydrorhodamine-123 (DHR; Sigma-Aldrich, St. Louis, MO, USA) was prepared in DMSO and stored at −20°C. This solution was diluted in HBSS to a 25 µg/ml working solution and stored in the dark until use. For ROS production, leukocytes (2×10^6^ cells) were incubated at 37°C with 2.5 µg DHR alone (negative control) or DHR + *R. equi* for 30 min; 80 ng of phorbol 12-myrisate 13-acetate (PMA) was used as a positive control.

The number of reactive neutrophils and mean fluorescent intensity was analyzed using a FACScan flow cytometer equipped with a 488-µm argon laser and Cell Quest Analysis Software (Beckton Dickinson, San Jose, CA, USA). Leukocyte subpopulations were displayed in a dot plot and gated according to size and granularity. A total of 10,000 data events were collected.

### Data analysis

For each cytokine, exploratory data analysis was performed. When the expression data were not normally distributed, logarithmic (log_10_) transformation was performed to approximate a normal distribution, as assessed using the Shapiro–Wilk test statistic. Linear mixed-effects modeling was used for analysis of all mRNA expression data, with individual foal modeled as a random effect to account for repeated measures over time (ages), and age was modeled as a fixed, ordered categorical effect. Linear mixed-effects modeling also was used for analysis of the degranulation or ROS data, with individual foal modeled as a random effect to account for repeated measures over time (ages), stimulus modeled as a categorical variable (with negative control as the reference group) and age modeled as a fixed, ordered categorical effect. Post hoc testing among times or stimuli was performed using the method of Sidak. A significance level of P<0.05 was used for all statistical analyses, which were performed using S-PLUS (version 8.1) statistical software (TIBCO, Seattle, WA, USA).

## Results

### Cytokine gene expression

**Figure 1:**
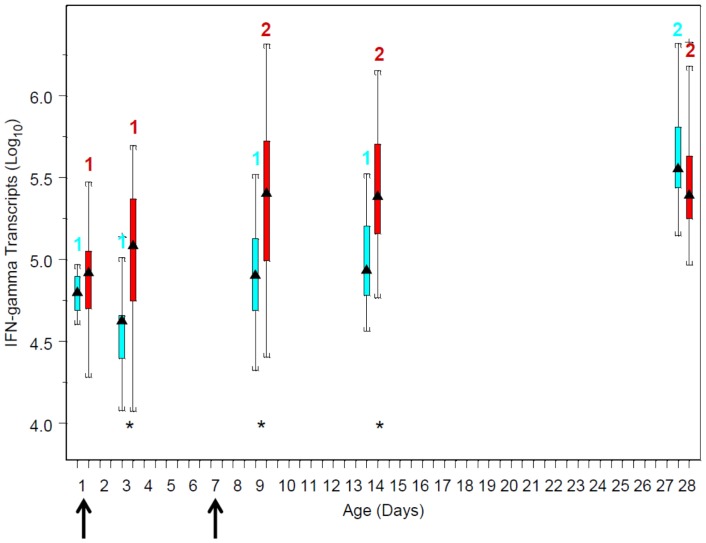
Boxplots of log_10_ values of mRNA copy numbers by age and treatment group: blue boxes are data from the control (saline) group and red boxes are data from the CpG-treated foals. The triangles in the middle of boxes represent the median value; the bottom and top of the boxes represent the 25^th^ and 75^th^ percentiles, respectively. The vertical lines extending from the boxes to horizontal lines represent multiples of 1.75 of the respective interquartile distance. Numbers above boxes represent within-treatment group differences among ages: within treatment group, ages with different numbers differed significantly (P<0.05) between groups. Asterisks denote days on which values were significantly (P<0.05) different between the control and Cp-G-treated foals. Arrows along the horizontal axis indicate ages when treatment (saline or CpG) was administered.

The expression of IFN-γ mRNA by neutrophils in response to *R. equi* stimulation *in vitro* was significantly (P<0.05) associated with age, and this effect was modulated by CpG-ODN treatment (Figure 1). For foals in both groups, IFN-γ mRNA expression in response to *R. equi* increased with age. For foals in the CpG group, values on days 1 and 3 were significantly (P<0.05) less than those on days 9, 14, and 28. For foals in the control group, however, values were only significantly different between day 28 and other days. At age 1 day, there was no significant difference in *R. equi*-stimulated IFN-γ expression between foals in the 2 groups; however, at ages 3, 9, and 14 days, IFN-γ mRNA expression was significantly higher in the CpG group; by 28 days, there was no significant difference between groups (Figure 1). Effects of treatment depended on age: at day 1 and by day 28, there were no significant differences between groups; however, on days 3, 9, and 14, foals in the CpG group had significantly (P<0.05) greater stimulated mRNA expression of IFN-γ mRNA (Figure 1).

For IL-4, absolute copy numbers were low at all ages (Table 1). Nevertheless, values of *R. equi*-induced significantly greater IL-4 on days 3, 9, 14, and 28 than on day 1 for foals in both groups; however, there were no significant effects of treatment on stimulated IL-4 mRNA expression between groups at any age (Table 1). There were no significant effects of age or treatment on expression of *R. equi*-stimulated mRNA expression of IL-6 or IL-8 (Table 2).

**Table 1:** Mean (and 95% confidence intervals) for mRNA copy numbers estimated by mixed-effects modeling for IL-4, IL-6, and IL-8.

Cytokine	Treatment Group	Day 1	Day 3	Day 9	Day 14	Day 28
IL-4	*Control*	2 (1–5)^a,1^	11 (4–28)^a,2^	14 (6–35)^a,2^	25 (10–62)^a,2^	22 (9–56)^a,2^
	*CpG*	4 (2–12)^a,1^	25 (11–60)^a,2^	32 (14–76)^a,2^	57 (24–135)^a,2^	52 (21–131)^a,2^
IL-6	*Control*	871^a,1^ (564–1,346)	1,256^a,1^ (816–1,934)	859^a,1^ (565–1,307)	888^a,1^ (581–1,358)	1,046^a,1^ (689–1,587)
	*CpG*	1,041^a,1^ (688–1,575)	1,501^a,1^ (993–2,272)	1,027^a,1^ (679–1,554)	1,060^a,1^ (701–1,603)	1,250^a,1^ (826–1,891)
IL-8	*Control*	169,824^a;1^ (83,992–343,368)	162,181^a,1^ (111,310–236,300)	126,882^a,1^ (88,032–182,877)	187,932^a,1^ (129,802–272,095)	172,187^a,1^ (119,465–248,176)
	*CpG*	389,942^a,1^ (164,679–923,336)	371,535^a,1^ (156,906–879,752)	290,670^a,1^ (122,755–688,272)	430,527^a,1^ (181,819–1,019,436	394,457^a,1^ (166,587–934,028)

Data represent back-transformed results of log_10_-transformed data. Values in columns with the same letter indicate absence of statistical significance between groups for a given age. Values in rows with differing superscripted numbers indicate significant (P<0.05) differences among ages within group.

**Table 2:** Mean (and 95% confidence intervals) for neutrophil parameters determined using flow cytometry (please see text for details) and estimated by mixed-effects modeling.

Neutrophil Parameter	Treatment Group	Day 1	Day 3	Day 9	Day 14	Day 28
Neutrophils	*Control*	7,100^a,1^ (6,627–7,773)	5,934^a,2,3^ (5,202–6,666)	6,419^a,1,2^ (5,684–7,154)	5,620^a,2,3^ (4,895–6,345)	5,138^a,3^ (4,413–5,862)
	*CpG*	6,933^a,1^ (6,225–7,640)	5,767^a,2,3^ (5,059–6,474)	6,252^a,1,2^ (5,544–6,959)	5,454^a,2,3^ (4,745–6,160)	5,515^a,3^ (4,807–6,222)
Reactive Neutrophils	*Control*	3,711^a,1^ (2,475–4,946)	1,580^a,2^ (154–3,006)	3,078^a,1^ (1,626–4,529)	2,871^a,1,2^ (1,445–4,297)	2,351^a,1,2^ (974–3,728)
	*CpG*	4,159^a,1^ (2,977–5,341)	2,028^a,2^ (846–3,210)	3,526^a,1^ (2,344–4,708)	3,319^a,1,2^ (2,137–4,501)	2,799^a,1,2^ (1,617–3,981)
Mean Neutrophil Fluorescence	*Control*	1,257^a,1^ (645–1,868)	1,400^a,1^ (676–2,124)	911^a,1^ (174–1,648)	1,534^a,1^ (836–2,233)	1,142^a,1^ (576–1,707)
	*CpG*	1,142^a,1^ (576–1,707)	1,285^a,1^ (719–1,850)	796^a,1^ (230–1,361)	1,389^a,1^ (823–1,954)	1,419^a,1^ (853–1,984)

Values in columns with the same letter indicate absence of statistical significance between groups for a given age. Values in rows with differing superscripted numbers indicate significant (P<0.05) differences among ages within group.

### Degranulation

**Figure 2:**
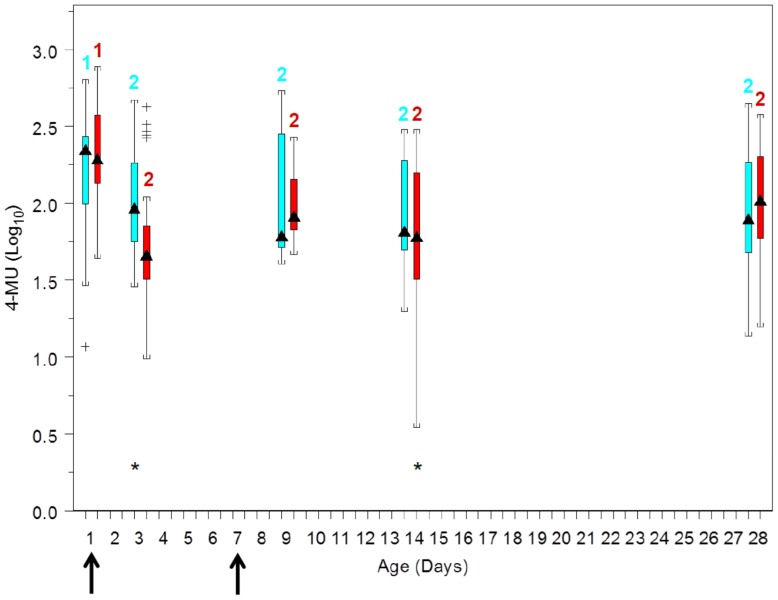
Boxplots of values of liberated 4-methylumbelliferone (4-MU) measured fluorometrically by age and treatment group: blue boxes are data from the control (saline) group and red boxes are data from the CpG-treated foal (see **Figure 1** for boxplot description). Numbers above boxes represent within-treatment group differences among ages: within treatment group, ages with different numbers differed significantly (P<0.05) between groups. Asterisks denote days on which values were significantly (P<0.05) different between the control and Cp-G-treated foals. Arrows along the horizontal axis indicate ages when treatment (saline or CpG was administered.

Degranulation responses decreased significantly (P<0.05) after day 1 for foals of both groups (Figure 2). The effects of age varied by treatment group: 4-methylumbelliferone concentration, an indicator of degranulation, was significantly (P<0.05) lower for foals in the CpG-ODN-treated group than the control group at days 3 and 14, but not at other days.

### ROS generation

Incubation with *R. equi* stimulated significant (P<0.0001) increases, relative to unstimulated cells, in mean fluorescence and in the number of reactive neutrophils. The number of neutrophils in samples decreased with age, but there was no significant effect of treatment on these age-related decreases (Table 2). The number of reactive neutrophils also tended to decreased with age (Table 2), but only values on day 3 differed significantly from those observed on day 1, and there were no significant effects of age (Table 2). There were no effects of age or treatment on the mean fluorescence of neutrophils (Table 2).

## Discussion

Results of our study substantiate previous findings that blood leukocytes in general [36]–[39] and neutrophils in particular [22–24) express less IFN-γ than older foals. It is plausible that reduced IFN-γ expression might diminish innate and adaptive immune responses of neonatal foals, thereby rendering them more susceptible to infectious diseases, and there is precedent for this in other species. For example, reduced IFN-γ expression by human neonatal cord blood monocytes in response to stimulation with respiratory syncytial virus was associated with increased cumulative incidence of viral respiratory tract disease during the first year of life [40]). Moreover, pre-treatment of neonatal mice with CpG-ODNs can increase IFN-γ by circulating neonatal natural killer (NK) cells, which are effector cells of innate immune responses [41]. Expression of IFN-γ by cells of the innate immune system can contribute to driving Th-1-type immune responses that are important for protecting against intracellular infections [42].

Innate immune responses to infection are important in neonates for at least 2 reasons. First, neonates are generally immunologically naïve at birth, and adaptive immune responses require time for development [10]–[12]. Second, innate responses are the first-line of defense against invading pathogens [10]–[12], [42]. Consequently, newborns rely heavily on innate immune responses for protection against infection. Neutrophils play a crucial role in innate immune responses: they are the most abundant immune cells in circulation and are generally the first immune effector cells to arrive at sites of infection. In addition to their primary bactericidal role, neutrophils can produce cytokines that may influence the development of adaptive immune responses [11], [42]. Thus, production of IFN-γ by neutrophils may contribute to limiting infection and enhancing responses to vaccines. Infectious diseases are leading causes of disease and death in foals [8], [9]. Clinical evaluation of the role of CpG-ODNs for protecting neonatal foals against infectious diseases is consequently warranted on the basis of the results of this study. Many important details regarding dosing will need to be considered for clinical use, including the number of doses administered. In this study, the CpG-ODN preparation was administered to foals at ages 1 and 7 days. The effects of the CpG-ODN preparation were apparent for at least 7 days after the second dose; however, effects appeared to have dissipated by 28 days of age. The duration of effects between 15 and 28 days of age of the regimen used in this study remains undefined. Thus, further study will be needed to characterize the duration of effects of the CpG-ODN and the number of doses tailored to the critical period for protection of various infections. The period of early life was of particular interest to us.

Although treatment had no effect on IL-4 mRNA expression by neutrophils, expression of this cytokine increased significantly between the day of birth and older ages. The clinical relevance of this finding is unknown. Interleukin-4 is considered to be a prototypical T helper (T_H_) 2-type cytokine, and neutrophils are known to produce IL-4 in low amounts [43]. It has been proposed that because neutrophils express the IL-4 receptor, they might be responsive even to low levels of IL-4 [43], as was observed in this study. Effects of IL-4 on neutrophils include stimulating differentiation, activation, protein expression, and, in an inflammatory milieu, enhanced respiratory burst activity and phagocytosis [44]–[46].

Irrespective of treatment group, we observed that degranulation appeared to be significantly higher on day 1 after birth relative to all other ages. The clinical importance of this result is unknown, but these results appear to suggest that neutrophils from 1-day-old foals are more prone to degranulation. Degranulation is a functional response of neutrophils that plays an important role in host defenses against microorganisms. A wide range of antimicrobial and potentially cytotoxic substances are contained within neutrophil granules, including antimicrobial proteins, proteases, components of the oxidative respiratory burst, membrane-bound receptors for endothelial adhesion molecules, extracellular matrix proteins, and soluble mediators of inflammation [44], [47]. Following degranulation, these substances are released to the phagosome or to the exterior of the cell where they exert antimicrobial effects. The activity of the released enzymes, such as myeloperoxidase, elastase, and β-D-glucuronidase (the azurophilic granule enzyme marker used in this study), can be used to monitor neutrophil degranulation. Conceivably, neutrophils of 1-day-old foals being more prone to degranulation could be a host defense mechanism designed to enhance innate immune responses of newborn foals. Alternatively, the tendency to more readily degranulate might represent a less mature, potentially noxious response that predisposes newborn foals to exaggerated responses to pathogens with deleterious results from releasing more granular contents.

There were small and inconsistent but significant effects of the administration of CpG-ODN 2142 on the degranulation of foal neutrophils: although degranulation responses were similar among foals in both treatment groups on day 1 of life, degranulation was significantly less on days 3 and 14 for foals treated with CpG-ODN 2142 compared to the control group. The interpretation of these findings is unclear. Conceivably, treatment with the CpG-ODN resulted in a more mature degranulation profile (i.e., less like that of 1-day-old foals); teleologically, this might result in less neutrophil-induced damage to the host's own tissues in response to invading micro-organisms. The magnitude of these effects were small, however (particularly on day 14), and were not observed on day 9. Thus, the clinical relevance of this observation remains unclear. We used *R. equi* as a stimulus for degranulation, based on previous results indicating that N-formyl-methionyl-leucyl-phenylalanine (fMLP) was not effective at stimulating degranulation in equine degranulation (20) and unpublished data from our laboratory documenting that *R. equi* was an effective stimulus of degranulation of foal neutrophils. It is possible that our findings would have differed had we used either another stimulus for degranulation or a different indicator of degranulation than β-D-glucuronidase activity. Interestingly, *in vitro* stimulation with CpG-ODN 2142 resulted in increased degranulation by neonatal foal neutrophils [20]. The reason for this discrepancy is unclear but may reflect either differences between *in vitro* and *in vivo* administration of CpG-ODNs, or the effects of the emulsifying adjuvant.

There were no significant effects of CpG-ODN administration on ROS production as measured by the number of reactive neutrophils or the mean fluorescence of neutrophils following *in vitro* stimulation with *R. equi*. Our laboratory previously described a priming effect of CpGs on *in vitro* stimulation of ROS generation in neutrophils stimulated with fMLP priming [24]. Thus, it was disappointing that *in vivo* administration of CpGs failed to enhance ROS production in response to *ex viv*o stimulation of neutrophils with *R. equi*. The observation that neutrophil concentrations decreased with age during the first month of life is well established [48]. Thus, it was not surprising that the total number of reactive neutrophils was significantly lower on day 3 than day 1, and tended to be lower for other days. Conceivably, the finding that the decrease in total neutrophils appeared progressive with age whereas the number of reactive neutrophils did not could represent a concomitant decrease in total numbers and an increase in functional response with age.

This study had a number of limitations. We did not document whether the increased expression of neutrophil IFN-γ had any effect on host susceptibility to infection. Foals in this study remained healthy throughout the project. A larger study using foals with either natural or experimental infections is needed to assess the role of CpG-ODN-induced expression of IFN-γ by neutrophils for protecting foals against infections. Such a study is warranted because evidence exists to suggest that production of IFN-γ by neutrophils could protect against infection. Neutrophils producing IFN-γ have been demonstrated in mice during infection with *Salmonella*, another intracellular pathogen [49]. Moreover, neutrophil-derived IFN-γ plays a role in protecting mice against *Listeria monocytogenes*, a facultative, intracellular pathogen that infects macrophages and other non-phagocytic cells [50]. Intra-tracheal administration of CpG-ODNs has been demonstrated to stimulate innate immune responses, including IFN-γ expression and IFN-γ-mediated processes that protected mice against experimentally-induced *Klebsiella* pneumonia [51].

Another limitation of our study is that we examined only the combination of CpG-ODN 2142 with Emulsigen but did not examine the effects of this CpG-ODN or the emulsifier independently. Thus, we cannot be sure whether the observed effects were attributable to the CpG-ODN alone, the emulsifying adjuvant, or both. Our rationale for examining the combination was 3-fold. First, we have previously documented *in vitro* that CpG-ODN 2142 influences neutrophil function of neonatal foals [23]. Second, there is extensive literature documenting enhanced *in vitro* and *in vivo* activity of CpG when combined with Emulsigen that is not attributable solely to the emulsifying adjuvant [25]–[35]. For example, Alves *et al.* documented that administration *in vivo* to pigs of CpG-ODN 2142 formulated with Emulsigen significantly increased gene expression of interferon-responsive genes by peripheral blood mononuclear cells relative to CpGs formulated with phosphate buffered saline or a double-oil emulsifier, or the adjuvant Montanide ISA 206 [25]. As another example, Ioannu *et al.* demonstrated that a bovine herpesvirus-1 subunit vaccine generated significantly greater virus-neutralizing antibodies and significantly greater protection against viral challenge than when the vaccine was formulated with either Emulsigen alone or CpGs alone [26]. Although CpG-ODNs are known to be potent activators of innate immune responses, their effects are often short-lived [34]. Emulsigen is considered to create a depot at the site of injection resulting in prolonged release of the CpG-ODNs from the injection site, thereby extending the immune-activating effects of CpG-ODNs in the host [26], [34]. Because of the improved immune activation from formulating CpG-ODNs with Emulsigen, it seemed most clinically relevant and appropriate to examine the combination. Third, we lacked an adequate population of foals necessary to allow for adequate statistical power to permit comparisons among 4 treatment groups (viz., CpG-ODN 2142 alone, Emulsigen alone, Emulsigen + CpG-ODN 2142, and saline [negative] control).

Despite these limitations, we believe the results of this study are promising. Intramuscular administration of newborn foals with CpG-ODN 2142 in combination with Emulsigen on the day of birth and 1 week later resulted in a significant increase in *R. equi*-stimulated IFN-γ expression. Moreover, the CpG-ODN with Emulsigen also appeared to modulate degranulation responses. Further evaluation of the impact of these functional responses on the susceptibility of neonates to infection and their ability to respond to vaccines is warranted because infectious diseases are major causes of disease and death in foals and other neonates [8], [9].

## Supporting Information

Table S1 Primer and probe sequences for amplification of various equine cytokines and β2-microglobulin.
(DOCX)
