# Supplementary material for: Intramuscular Administration of a Synthetic CpG-Oligodeoxynucleotide Modulates Functional Responses of Neutrophils of Neonatal Foals
Source: PLoS One. 2014 Oct 15;9(10):e109865. doi: 10.1371/journal.pone.0109865 (PMC4198146; doi:10.1371/journal.pone.0109865)
Supplement: Table S1 — Primer and probe sequences for amplification of various equine cytokines and β2-microglobulin. (DOCX) [file pone.0109865.s001.docx]

**Table S1.** Primer and probe sequences for amplification of various equine cytokines and β2-microglobulin.

| Gene | Primer/probe | Sequence 5’-3’ |
| --- | --- | --- |
| IFN-γ | Forward | AAGTGAACTCATCAAAGTGATGAATGA |
|  | Reverse | CGAAATGGATTCTGACTCCTCTTC |
|  | Probe | TCGCCCAAAGCTAACCTGAGGAAGC |
| IL-4 | Forward | TGAGCGGACTGGACAGGAA |
|  | Reverse | CCAAAAAGTCTTTCAATGTGCTC |
|  | Probe | AGGTCCCGTTTGCCATGCCCTT |
| IL-6 | Forward | GAAAAAGACGGATGCTTCCAATCTG |
|  | Reverse | TCCGAAAGACCAGTGGTGATTTT |
|  | Probe | CAGGTCTCCTGATTGAAC |
| IL-8 | Forward | GCCACACTGCGAAAACTCA |
|  | Reverse | GCACAATAATCTGCACCCACTTTG |
|  | Probe | ACGAGCTTTACAATGATTTC |
